# Aedes albopictus in the United States: ten-year presence and public health implications.

**DOI:** 10.3201/eid0303.970309

**Published:** 1997

**Authors:** C G Moore, C J Mitchell

**Affiliations:** Centers for Disease Control and Prevention, Fort Collins, Colorado 80522-2087, USA. cgm2@cdc.gov

## Abstract

Since its discovery in Houston, Texas, in 1987, the Asian "tiger mosquito" Aedes albopictus has spread to 678 counties in 25 states. This species, which readily colonizes container habitats in the peridomestic environment, was probably introduced into the continental United States in shipments of scrap tires from northern Asia. The early pattern of dispersal followed the interstate highway system, which suggests further dispersal by human activities. The Public Health Service Act of 1988 requires shipments of used tires from countries with Ae. albopictus to be treated to prevent further importations. Given the extensive spread of the mosquito in the United States, it is questionable whether such a requirement is still justified. Ae. albopictus, a major biting pest throughout much of its range, is a competent laboratory vector of at least 22 arboviruses, including many viruses of public health importance. Cache Valley and eastern equine encephalomyelitis viruses are the only human pathogens isolated from U.S. populations of Ae. albopictus. There is no evidence that this mosquito is the vector of human disease in the United States.


### *Aedes albopictus* in the United States: Ten-Year Presence and Public Health Implications

**Chester G. Moore and Carl J. Mitchell**
Centers for Disease Control and Prevention,
Fort Collins, Colorado, USA

Since its discovery in Houston, Texas, in 1987, the Asian "tiger mosquito" *Aedes albopictus* has spread to 678 counties in 25 states. This species, which readily colonizes container habitats in the peridomestic environment, was probably introduced into the continental United States in shipments of scrap tires from northern Asia. The early pattern of dispersal followed the interstate highway system, which suggests further dispersal by human activities. The Public Health Service Act of 1988 requires shipments of used tires from countries with *Ae. albopictus* to be treated to prevent further importations. Given the extensive spread of the mosquito in the United States, it is questionable whether such a requirement is still justified. *Ae. albopictus*, a major biting pest throughout much of its range, is a competent laboratory vector of at least 22 arboviruses, including many viruses of public health importance. Cache Valley and eastern equine encephalomyelitis viruses are the only human pathogens isolated from U.S. populations of *Ae. albopictus.* There is no evidence that this mosquito is the vector of human disease in the United States.

Established populations of *Aedes albopictus*, the Asian "tiger mosquito," (1) were first discovered in the continental United States in Harris County, Texas, in August 1985 (2). (*Ae. albopictus* was introduced into Hawaii sometime before 1902 (3). This mosquito may have become established in the region even earlier since an adult female was collected in Memphis, Tennessee, in 1983 (4). *Ae. albopictus* probably entered the United States in shipments of used tires from northern Asia, where the species is widely distributed (5-7). Beginning January 1, 1988, the U.S. Public Health Service required that all used tires arriving at U.S. ports from areas known to be infested with *Ae. albopictus* be dry, clean, and fumigated or otherwise "disinsected" (8). However, by the time the disinsection requirement was put in place, existing populations had become established in 15 states.

*Ae. albopictus* is both a nuisance and a potential disease vector. Anecdotal reports from local mosquito control agencies suggest that it has become a major pest mosquito problem in many communities in the southeastern United States. Laboratory studies show that this species is susceptible to and can transmit many arboviruses of public health importance (9-12). In this article, we summarize the reported distribution and dispersal of *Ae. albopictus* in the past 10 years and review surveillance for infection and transmission of arboviruses.

Address for correspondence: Chester G. Moore, Ph.D., Division of Vector-Borne Infectious Diseases, National Center for Infectious Diseases, CDC, P.O. Box 2087, Fort Collins, CO 80522-2087, USA; fax: 970-221-6476; e-mail: cgm2@cdc.gov.

### Distribution and Dispersal of *Aedes albopictus*

A national database of the distribution of *Aedes albopictus* is maintained as a passive surveillance system (13); the system is periodically stimulated by letters and telephone calls to mosquito and vector control professionals throughout the United States, as well as by articles in professional journals and newsletters and presentations at professional meetings. Data obtained from a standardized reporting form sent to potential collaborators to ensure standardization of the data are entered into a computerized database written in EpiInfo (14). Summary data are extracted from the database for reports or for transfer to a desktop mapping program.

### Temporal Patterns

Figure 1 shows the changing distribution of *Ae. albopictus* over time. The mosquito is widely distributed in the southeastern United States. Established infestations are less common northward and westward, presumably because of less hospitable environments. The absence of reports from some states may reflect lack of surveillance rather than absence of the mosquito. This species may have been present in some areas for many years before discovery (particularly true in areas without active mosquito surveillance or control programs). In certain states, such as South Carolina and Kentucky, the abrupt discovery of *Ae. albopictus* in a large number of counties was the result of university graduate students' research.

### Possible Dispersal Routes

During the early period of dispersal, the presence of *Ae. albopictus* appeared to be related to the proximity of a county to interstate highways (Figure 2). In December 1987, 92 counties in 15 states were infested with this mosquito. Of the 1,511 counties in states where *Ae. albopictus* was present, 582 (38.5%) had interstate highways passing through them. Were the spread of *Ae. albopictus* not related to the interstate system, only 35 (38%) of the 92 mosquito-infested counties would be expected to lie on an interstate highway. In fact, 64 of the 92 infested counties were on an interstate ($X^2 = 25.29$, df = 3, $p < 0.001$).

The postulated relationship between dispersal and major transportation routes would be expected for a species transported largely by human activities such as the commercial movement of scrap tires for retreading, recycling, or other purposes. Several of the 28 mosquito-infested sites not located on the interstate system were major tire retreading companies, other businesses that deal with large numbers of used or scrap tires, or illegal tire dumps.

Once populations of the mosquito become established, local transport and active migration should disperse the mosquito throughout the surrounding area. As would be expected if the original infestation were in Texas, the proportion of *Ae. albopictus*-infested counties on the interstate system in Texas had fallen from 1 (100%) of 1 in 1985, to 13 (65%) of 20 in 1986, and to 23 (58%) of 39 in 1987.

This distribution pattern can be explained in other ways. At least early on, searches might have been limited to *Ae. albopictus* in the major cities. Since most major cities are connected by interstates, it is impossible to separate these two possibilities. Moreover, most of the active vector control programs and other activities that would involve surveys for this mosquito are probably located in larger cities, which are connected to the interstate highway system.

### *Ae. albopictus* as a Disease Vector

### Vector Competence Studies

Reviews of many vector competence studies involving *Ae. albopictus* (9-12) provide information for 23 arboviruses and for Nodamura virus (probably not an arbovirus). In addition, *Ae. albopictus* has been recently found to be a competent experimental vector of Sindbis virus (15). A list of viruses included in vector competence experiments involving *Ae. albopictus* is shown in Table 1.

*Ae. albopictus* is a competent experimental vector of seven Alphaviruses: Chikungunya, eastern equine encephalitis (EEE), Mayaro, Ross River, western equine encephalitis, Venezuelan equine encephalitis, and Sindbis viruses. Only EEE virus has been isolated from *Ae. albopictus* collected in nature.

*Ae. albopictus* is also a competent experimental vector of the following Flaviviruses: dengue (DEN) serotypes 1, 2, 3, and 4, Japanese encephalitis, West Nile, and yellow fever viruses. In the case of an additional Flavivirus, St. Louis encephalitis virus, the amount of circulating virus in naturally infected avian hosts is generally insufficient to infect the mosquito (16).

DEN and Japanese encephalitis viruses have been isolated from specimens of *Ae. albopictus* collected outside the United States, and these viruses can be transmitted vertically under experimental conditions (9). Recently, isolation of DEN-1 virus from *Ae. albopictus* larvae in Brazil has been reported (17). *Ae. albopictus* has been involved in the transmission of DEN viruses in southeast Asia, southern China, Japan, and the Seychelles (18). If DEN viruses were introduced into areas of the United States with dense populations of *Ae. albopictus*, this mosquito could conceivably act as a vector. However, the classic epidemic vector of DEN viruses, *Ae. aegypti*, is also present in many of the southeastern states; in areas where *Ae. aegypti* is abundant, this species might be expected to play a far more important role in DEN transmission than *Ae. albopictus*.

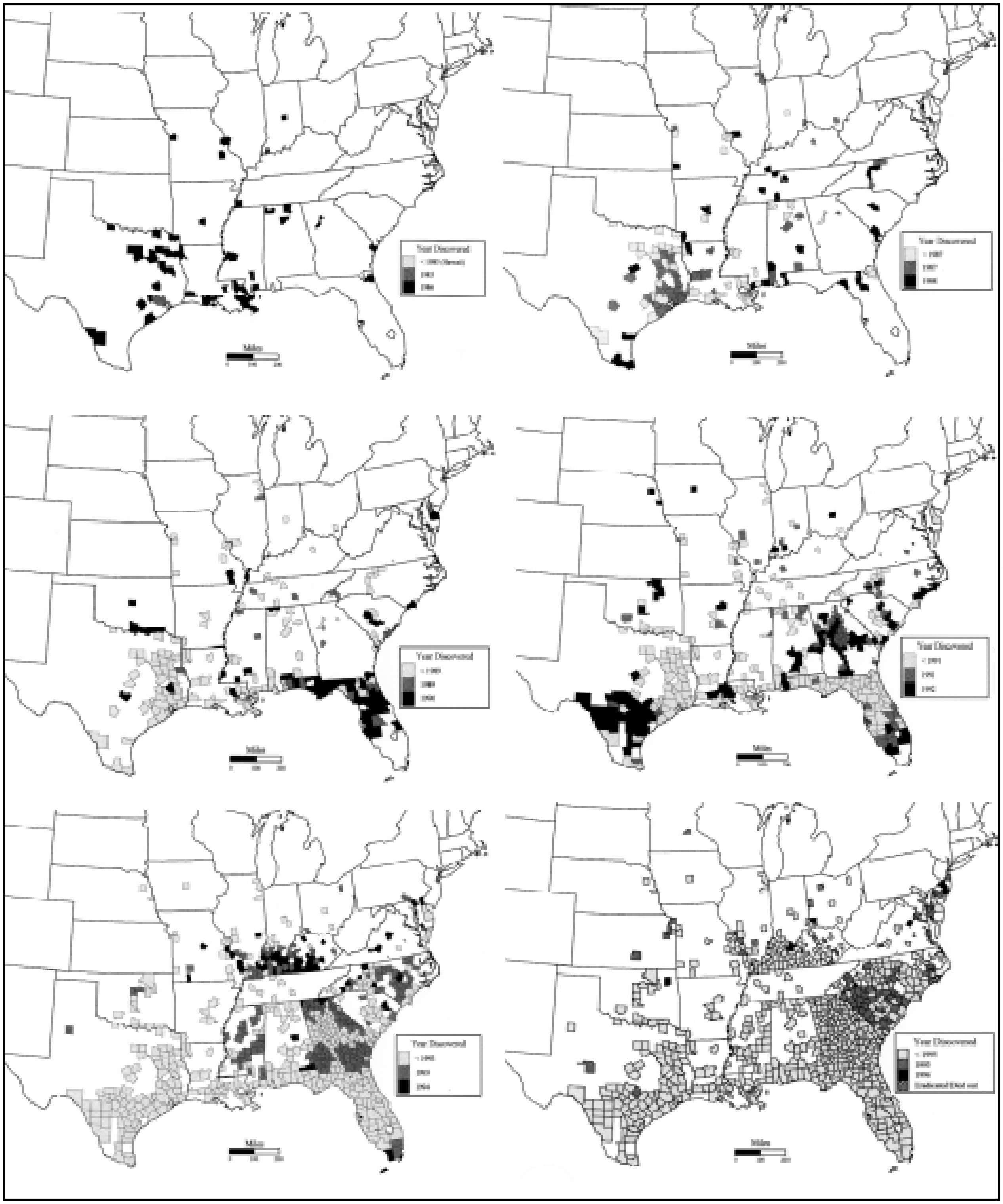

Figure 1. Reported distribution of *Aedes albopictus*, the Asian "tiger mosquito," in the continental United States, 1985-1996. Maps were generated by merging the EpiInfo database into the Atlas geographic information system.

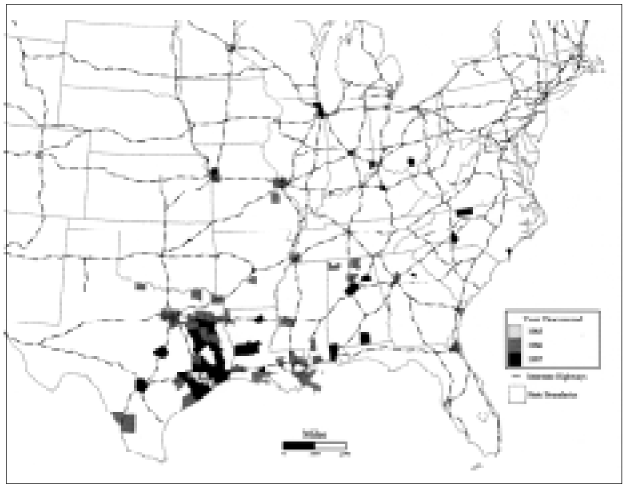

Figure 2. Apparent relationship between the early dispersal of *Aedes albopictus* and the U.S. interstate highway system, 1985-1987. Map generated by merging EpiInfo database into the Atlas geographic information system.

Vector competence tests show that eight Bunyaviridae (Jamestown Canyon, Keystone [KEY], LaCrosse, Oropouche, Potosi, Rift Valley fever, San Angelo, and trivittatus viruses) infect *Ae. albopictus* by the oral route. Only the KEY, Oropouche, and trivittatus viruses are not transmitted efficiently by bite. The KEY, LaCrosse, and San Angelo viruses can be transmitted vertically under experimental conditions.

### Field Investigations

Since the discovery of *Ae. albopictus* in the United States, field-collected *Ae. albopictus* from several areas have been tested for arboviruses. From 1987 to 1995, 122,879 specimens were tested from 12 states (Table 2). Four viruses have been isolated: Potosi (19-22), EEE (23), KEY (23, 24 and R. Nasci, unpub. data), and Cache Valley (CV) virus (CDC, unpub. data). Tensaw virus was isolated by the Texas State Department of Health (23). The geographic and temporal distributions of these virus isolations are shown in Table 3.

Aside from EEE and CV viruses, the viruses isolated from *Ae. albopictus* in the United States are not of public health importance. The association of *Ae. albopictus* with EEE virus in nature has been restricted to a single incident in Polk County, Florida, in 1991. The presence of a large tire dump (ca. 1.5 million used tires) within a known enzootic focus of EEE virus (the Green Swamp) may have led to an unusual virus-vector association (23). Follow-up studies at the dump site in 1992, after the tires had been shredded, yielded fewer than 1,000 *Ae. albopictus* (none infected) (Mitchell and Niebylski, pers. comm.), and EEE virus has not been isolated from field-collected specimens of this species since the original episode. CV virus was isolated from a pool of *Ae. albopictus* collected in Jasper County, Illinois, in 1995 (CDC, unpub. data). During the same year, a case of human disease with diverse clinical manifestations due to CV virus was reported in a patient who presumably contracted the infection while deer hunting in Anson County, North Carolina (25). However, there is little reason to suspect that *Ae. albopictus* was involved in this incident. CV virus was isolated repeatedly from several other genera and species of mosquitoes before *Ae. albopictus* was present in the continental United States and, thus far, CV virus has been isolated from *Ae. albopictus* from only a single pool of specimens in Illinois.

Table 1. Susceptibility of *Aedes albopictus* to oral infection with arboviruses and ability to transmit by bite*

| | *Ae. albopictus* strains | | | |
|---|---|---|---|---|
| | Hawaii and areas outside W. Hemisphere | | North and South America | |
| Viruses | Infect. | Trans. | Infect. | Trans. |
| Chikungunya | + | + | + | + |
| Dengue 1, 2, 3, 4 | + | + | + | + |
| Eastern equine encephalitis | + | + | + | + |
| Jamestown Canyon | | | + | + |
| Japanese encephalitis | + | + | | |
| Keystone | | | + | - |
| La Crosse | | | + | + |
| Mayaro | | | + | + |
| Nodamura | + | ? | | |
| Oropouche | | | + | - |
| Orungo | + | + | | |
| Potosi | | | + | + |
| Rift Valley fever | | | + | + |
| Ross River | + | + | + | + |
| San Angelo | + | + | | |
| Sindbis | | | + | + |
| St. Louis encephalitis | + | + | | |
| Trivittatus | | | + | - |
| West Nile | + | + | | |
| Western equine encephalitis | + | + | + | + |
| Venezuelan equine encephalitis | | | + | + |
| Yellow fever | + | + | + | + |

* Modified from Mitchell (1991)(10)

Table 2. Field-collected *Aedes albopictus* tested for virus, 1987-1995*

| State of origin | Number tested |
|---|---|
| Alabama | 64 |
| Arkansas | 1,234 |
| Florida | 18,862 |
| Illinois | 10,921 |
| Indiana | 516 |
| Louisiana | 47,320 |
| Mississippi | 128 |
| Missouri | 35,797 |
| North Carolina | 4,590 |
| Ohio | 1,604 |
| South Carolina | 72 |
| Tennessee | 1,771 |
| TOTAL | 122,879 |

*Tests were conducted by the Centers for Disease Control and Prevention

Table 3. Arboviruses isolated from *Aedes albopictus* in the United States, 1987-1996*

| Virus | State | County or parish | Year |
|---|---|---|---|
| Potosi | Missouri | Washington | 1989 |
| | North Carolina | Anson | 1994 |
| | Illinois | Jasper | 1994 |
| Eastern equine encephalitis | Florida | Polk | 1991 |
| Keystone | Florida | Polk | 1991 |
| | Florida | Orange | 1993 |
| | Louisiana | Calcasieu | 1995 |
| Tensaw | Texas | Montgomery | 1991 |
| Cache Valley | Illinois | Jasper | 1995 |

*All viruses except Tensaw were isolated in the CDC laboratory in Fort Collins, Colorado; Tensaw virus was isolated by the Texas State Health Department, Austin, Texas.

### Conclusions

*Ae. albopictus* is firmly established in the United States. In the 10 years since its discovery, this species has spread throughout much of the East. The species occurs in all counties in at least four states—Delaware, Florida (26), Georgia (27), and South Carolina. It probably occurs in all or most counties in Alabama, Mississippi, and Louisiana, but surveys from those states are incomplete. *Ae. albopictus* seems to be approaching the northern limit predicted by Nawrocki and Hawley (28). The westward dispersal of this mosquito has been very slow, perhaps because the drier environment of the Great Plains region inhibits westward movement of this species. Data suggest that the current practice of requiring the disinsection of used tires entering the United States from other countries with *Ae. albopictus* does not influence the dynamics and spread of this species within this country. If disinsection is to remain in force, other justification will be needed.

Observations on early dispersal of *Ae. albopictus* are consistent with the hypothesis of dispersal by human activities, probably movement of scrap tires through the interstate highway system. This information might be useful in designing monitoring programs for possible future introductions of mosquitoes.

Collectively, the above information indicates that *Ae. albopictus* is a competent vector for a wide variety of arboviruses under experimental conditions, has been found to be naturally infected with DEN, Japanese encephalitis, Potosi, KEY, Tensaw, CV, and EEE viruses, and can serve as an epidemic vector of DEN viruses. The capacity of *Ae. albopictus* to vertically transmit certain arboviruses may also enhance the possibility of establishing new enzootic and endemic foci of some viruses. *Ae. albopictus* is a major biting pest throughout much of its range and is of justifiable concern to mosquito control and public health agencies for this reason alone. Nonetheless, in terms of its role as an arbovirus vector, evidence is lacking to incriminate *Ae. albopictus* as the vector of even a single case of human disease in the United States.

### Acknowledgments

The *Aedes albopictus* distribution database is the result of the effort of nearly 100 colleagues in state and local health and vector control agencies, universities, and military agencies. We acknowledge K.W. Blank and G.M. Beavers, University of Kentucky, and D. Berge, University of South Carolina, for sharing unpublished data for the distribution maps.

### References

1. Robertson RC, Hu SMK. The tiger mosquito in Shanghai. The China Journal 1935;23:299-306.
2. Usinger, RL. Entomological phases of the recent dengue epidemic in Honolulu. Public Health Rep 1944;59:423-30.
3. Sprenger D, Wuithiranyagool T. The discovery and distribution of *Aedes albopictus* in Harris County, Texas. J Am Mosq Control Assoc 1986;2:217-9.
4. Reiter P, Darsie RF Jr. *Aedes albopictus* in Memphis, Tennessee (U.S.A.): an achievement of modern transportation? Mosquito News 1984;44:396-9.
5. Hawley WA, Reiter P, Copeland RW, Pumpuni CB, Craig, Jr GB. *Aedes albopictus* in North America: probable introduction in used tires from northern Asia. Science 1987;236:1114-6.

6. Reiter P, Sprenger D. The used tire trade: a mechanism for the worldwide dispersal of container breeding mosquitoes. J Am Mosq Control Assoc 1987;3:494-501.
7. Craven RB, Eliason DA, Francy DB, Reiter P, Campos EG, Jakob WL, et al. Importation of *Aedes albopictus* and other exotic mosquito species into the United States in used tires from Asia. J Am Mosq Control Assoc 1988;4:138-42.
8. Centers for Disease Control. Requirement of certification of used tire casings from Asia prior to entry into the United States. Federal Register 1987;52(224):44646.
9. Shroyer DA. *Aedes albopictus* and arboviruses: a concise review of the literature. J Am Mosq Control Assoc 1986;2:424-8.
10. Mitchell CJ. Vector competence of North and South American strains of *Aedes albopictus* for certain arboviruses. J Am Mosq Control Assoc 1991;7:446-51.
11. Mitchell CJ. Geographic spread of *Aedes albopictus* and potential for involvement in arbovirus cycles in the Mediterranean basin. Journal of Vector Ecology 1995;20:44-58.
12. Mitchell CJ. The role of *Aedes albopictus* as an arbovirus vector. Proceedings of the Workshop on the Geographic Spread of *Aedes albopictus* in Europe and the Concern among Public Health Authorities; 1990 Dec 19-20; Rome, Italy. Parassitologia; 1995;37:109-13.
13. Teutsch SM. 1994. Considerations in planning a surveillance system. In: Teutsch SM, Churchill RE, editors. Principles and practice of public health surveillance. New York: Oxford University Press; 1994. p. 18-30.
14. Dean AG, Dean JA, Coulombier D, Brendel KA, Smith DC, Burton AH, et al. Epi Info, Version 6: a word processing, database, and statistics program for epidemiology on microcomputers. Atlanta (GA): Centers for Disease Control and Prevention: 1994.
15. Dohm DJ, Logan TM, Barth JF, Turell MJ. Laboratory transmission of Sindbis virus by *Aedes albopictus*, *Ae. aegypti*, and *Culex pipiens* (Diptera: Culicidae). J Med Entomol 1995;32:818-21.
16. Savage HM, Smith GC, Mitchell CJ, McLean RG, Meisch MV. Vector competence of *Aedes albopictus* from Pine Bluff, Arkansas, for a St. Louis encephalitis virus strain isolated during the 1991 epidemic. J Amer Mosq Control Assoc 1994;10:501-6.
17. Serufo JC, Montes de Oca H, Tavares VA, Souza AM, Rosa RV, Jamal MC, et al. Isolation of dengue virus type 1 from larvae of *Aedes albopictus* in Campos Altos City, State of Minas Gerais, Brazil. Mem Inst Oswaldo Cruz 1993;88:503-4.
18. Hawley WA. 1988. The biology of *Aedes albopictus*. J Am Mosq Control Assoc 1988;4(Suppl. 1):1-40.
19. Francy DB, Karabatsos N, Wesson DM, Moore Jr CG, Lazuick JS, Niebylski ML, et al. A new arbovirus from *Aedes albopictus*, an Asian mosquito established in the United States. Science 1990;250:1738-40.
20. Mitchell CJ, Smith GC, Miller BR. Vector competence of *Aedes albopictus* for a newly recognized *Bunyavirus* from mosquitoes collected in Potosi, Missouri. J Am Mosq Control Assoc 1990;6:523-7.
21. Mitchell CJ, Smith GC, Karabatdsos N, Moore CG, Francy DB, Nasci RS. Isolations of Potosi virus from mosquitoes collected in the United States, 1989-1994. J Am Mosq Control Assoc 1996;12:1-7.
22. Harrison BA, Mitchell CJ, Apperson CS, Smith GC, Karabatsos N, Engber BR, et al. Isolation of Potosi virus from *Aedes albopictus* in North Carolina. J Am Mosq Control Assoc 1995;11:225-9.
23. Mitchell CJ, Niebylski ML, Smith GC, Karabatsos N, Martin D, Mutebi J-P, et al. Isolation of eastern equine encephalitis from *Aedes albopictus* in Florida. Science 1992;257:526-7.
24. Mitchell CJ, Morris CD, Smith GC, Karabatsos N, Vanlandingham D, Cody E. Arboviruses associated with mosquitoes from nine Florida counties during 1993. J Am Mosq Control Assoc 1996;12:255-62.
25. Sexton DJ, Rollin PE, Breitschwerdt EB, Corey GR, Myers SA, Hegarty BC, et al. Brief report: severe multiorgan failure following Cache Valley virus infection. N Engl J Med 1997;336:547.
26. O'Meara GF, Evans Jr LF, Gettman AD, Cuda JP. Spread of *Aedes albopictus* and decline of *Ae. aegypti* (Diptera: Culicidae) in Florida. J Med Entomol 1995;32:554-62.
27. Womack ML, Thuma TS, Evans BR. Distribution of *Aedes albopictus* in Georgia, USA. J Am Mosq Control Assoc 1995;11:237.
28. Nawrocki SJ, Hawley WA. Estimation of the northern limits of distribution of *Aedes albopictus* in North America. J Am Mosq Control Assoc 1987;3:314-7.
